# The Physician and His Microscope1Read at the nineteenth annual meeting of the American Microscopical Society, Pittsburg, Pa., August 19, 1896.

**Published:** 1896-10

**Authors:** A. A. Young

**Affiliations:** Newark, N. Y. 22 East Miller Street


### THE PHYSICIAN AND HIS MICROSCOPE.[1]

BY A. A. YOUNG, M. D., Newark, N. Y.

ONE of the most expensive and one of the most useless pieces of office furniture that the ordinary physician possesses is his microscope. It usually occupies a most commanding and conspicuous place in the office and decorated with "fuss and feathers"; valueless as an educator, valuable for the macroscopical appearances of the microscope, for it is capable of producing wonder and awe to the office visitor and shekels to the pocket of the physician.

Nothing can be said against the microscope as an instrument, for its value resides in its intelligent use, and unless used intelligently it becomes worse than useless, distorting facts and fancies alike, from which the observer can form no concept, can draw no conclusion save an erroneous one. The physician has to deal with the organic world, with those material forms in which resides that peculiar, unresolvable and unknowable agent we call life, and without which matter becomes comparatively valueless.

The microscope in the department of medicine requires for its intelligent manipulation a familiarity with anatomy, pathology, bacteriology, and last, but not least, biology, which subject scarcely ever enters into a medical college curriculum. We, as physicians, must deal with material forms that are endowed with life, and of that relation which exists between the material form and life we must have some concept, though it be partial and inadequate, for on the relation of things material or immaterial is the development of human thought possible. The life force of the bacillus is doubtless as intricate as the life force of the human subject and may be similar if not identical with it; for what is the body in which the ego resides more than an aggregation of amebæ specialised, and each ameba possibly having an independent life and having reproductive properties of its own. It is with the minute mass of matter, not the molecule, that the microscopist has to deal; he sees its manner and method of growth and not the forces which produce the molecular arrangement of the ultimate particles.

It is not enough that the physician be able to observe and differentiate the various forms of the micrococcus, spirillum or bacillus: he must know as well the habitat, manner and method of growth of each variety. Without this knowledge the revelations of the microscope are no more intelligible than some Egyptian

1. Read at the nineteenth annual meeting of the American Microscopical Society, Pittsburg, Pa., August 19, 1896.

inscriptions. There is a philosophy of microscopy which is equally as valuable as the facts on which it is based, but a philosophy that can only be developed by accurate observation and classification of microscopical data. This work, it is evident, must be performed by the skilled microscopist and not by the novice, in which class the busy practitioner is usually found. In microscopical analysis no element relative to accuracy can with safety be omitted. It matters not though the microscopical accessories be thoroughly cleansed and sterilised, for the results would be equally untrustworthy if the material to be examined be placed in a receptacle, found perhaps in some old garret and half cleansed. Conclusions reached under such conditions must be erroneous. Do you ask who ever allows such procedures ? Go to the home of the amateur or pseudo-microscopist, observe his methods and technique and you will have the answer. It is surprising how much we see, how much we assume and how little we know. A young physician asks an older one for the use of his microscope to examine a specimen of urine, assuring its owner that he is familiar with the instrument, having had instruction in college ; permission granted, and slide prepared, and the observer exclaims, " The most beautiful specimen of a cast I have ever seen ; " the owner of the instrument says, "That looks like vegetable matter and not a cast." "No," said the other, " that is a urinary cast ; I have seen many of them." A microscopical examination of the container and its contents revealed a corncob for a cork ; what the cast was you may readily infer.

A physician of several years' standing and the possessor of a good microscope at an autopsy of his announced that the patient's death was due to a disease of the kidneys, that she had been passing blood, pus, all forms of casts and other bad material with the urine. The autopsy, however, revealed ulceration with pus formation, degeneration and rupture of the gall-bladder, produced by impacted gall-stones, while the kidneys were practically normal, showing no structural degeneration. From whence, then, came the blood, pus, casts and débris, which was alleged to have been seen? These cases could have been none other than of mistaken identity ; something was inferred that did not exist.

The conclusion is therefore reached, justly or otherwise, that the eye and understanding must be educated independently along certain lines before the manipulation of the microscope becomes satisfactory and trustworthy ; objects must be seen and known relatively and in their entirety before being resolved into their component elements; the macroscopical appearance of an object must precede its microscopical appearance.

The physician must know in what menstruum and under what conditions the objects for which he is searching exists or are developed. Neither is it enough for him to know and recognise the various forms of bacilli; he must be able to classify them and know their manner and method of growth, what they produce by their growth and what influence they have upon humanity. This is the philosophy of microscopy as relates to medical science. The microscope therefore becomes to the physician valuable in the degree that he is able to classify and arrange its revelations so that they may be read as from an open book. This faculty means a familiarity with the instrument born of time,—time which the "country doctor" must give by piecemeal, if at all.

I am no pessimist, although I see in a degree the passing of the microscope so far as it relates to the individual work of the ordinary medical practitioner. As already intimated, this passing is induced and sustained by unskilled and untrained eyes, which see much and individualise little.

The structure of microscopy, if it be enduring, must be built upon a comparatively errorless macroscopy. The rank and file still have to learn that the microscope only enables the investigator to continue his eyesight so as to observe the primary structure of an organised mass that would otherwise remain unknown and unknowable.

The first essential, then, for a physician microscopist is the proper use of his eyes, supplemented by a keen intellect; what he sees he must be able to describe accurately, thus differentiating the various forms and figures that appear in the visual field.

Neither is it enough for him to recognise an object in an isolated condition and know its form and construction: he must know as well what relation it sustains to other objects about it. This calls for the exercise of the comparative faculty, the second essential for the physician microscopist; indeed, these two elements may be called his eyes. With these faculties undeveloped, untrained, he may as well be a blind microscopist. What is true of normal vision is preëminently true of aided vision, which aid the microscope is, but it produces changes also in the relative conditions of objects, and of such changes the mind must take cognisance; it is an element too often overlooked. In short, the revelations of the microscope becomes the alphabet and the systematic arrangement of these revelations in the human mind forms its language, a language that requires study to comprehend; a language also that needs much further development and amplification. Physicians, as a rule, can be novices only in microscopical science, following where others lead; they stand at your feet, at the feet of the microscopists of the world, in the relation of pupil to teacher, asking for more light to illuminate the intricacies of human existence.

Give to them this light; save for them the microscope with all of its powers and possibilities which are vast; prevent it by your efforts from relapsing into a state of "innocuous desuetude."

22 East Miller Street.

